# Experimental Study on the Application of Limestone Mine Dust Filter Slag as Concrete Admixture

**DOI:** 10.3390/ma18173970

**Published:** 2025-08-25

**Authors:** Yuehua Liang, Jie Wang

**Affiliations:** School of Civil and Architectural Engineering, Panzhihua University, Panzhihua 617000, China; 7120190003@mail.jxust.edu.cn

**Keywords:** supplementary cementitious materials, fly ash, activity index, flowability, hydration products, phase analysis

## Abstract

With rapid industrialization, large quantities of industrial solid waste are generated annually. In Panzhihua, China, approximately 300,000 tons of limestone mine dust filter residue (LMDFR) is produced. This study investigates the properties of LMDFR and its potential as a supplementary cementitious material. LMDFR was blended with fly ash (FA) to replace 30% of cement in mortar. Tests were conducted to measure the mortar’s flowability and its compressive and flexural strengths after 7 and 28 days of curing, and XRD, SEM, TG, and DSC analyses were conducted on 28-day specimens. LMDFR primarily comprises ≥95% CaCO_3_, with a specific surface area of ~1.3 m^2^/g and density of 2.694 g/cm^3^. Mortar flowability increased with LMDFR content, reaching 112.83% when used alone. Flexural strength was largely unaffected, while the 7-day compressive strength significantly improved. However, the 28-day strength decreased when LMDFR was used alone, with a 28-day activity index of 61.10%, compared with 71.52% for FA. A 1:1 blend of LMDFR and FA improved the activity index to 83.18%. Microstructural and thermal results corroborated strength and flowability trends. In conclusion, LMDFR demonstrates promising potential as a supplementary cementitious material in concrete applications. When blended with fly ash at a 1:1 ratio, the composite admixture significantly enhances flowability and early compressive strength while maintaining adequate long-term performance. This synergistic combination not only improves the physical properties of cement mortar but also provides a sustainable solution for the large-scale utilization of industrial solid waste.

## 1. Introduction

With the advancement of industrialization, vast quantities of industrial solid waste are being generated. By 2025, the cumulative global stockpile of bulk industrial solid waste is projected to exceed 60 billion tons, with China alone accounting for approximately 6 billion tons. The global annual generation of bulk industrial solid waste is expected to reach 30 billion tons, of which China contributes nearly 10%, or about 3 billion tons annually. By 2025, China’s comprehensive utilization rate of bulk industrial solid waste is anticipated to reach 57%, with the resource recycling industry achieving an annual output value of 5 trillion RMB. Industrial solid waste is typically handled through several pathways: (1) enrichment and extraction of valuable metals [1,2]; (2) conversion into green building materials [3,4]; (3) reuse in civil engineering applications [5,6]; and (4) mine backfilling [7,8].

In industrial processes, solid waste is often mixed with liquids to form sludge. Common treatment methods include sedimentation and pressure filtration to recover water for reuse in production and daily operations. The remaining filter residue is then disposed of or utilized as solid waste. For example, Nilanjan Santra et al. [9] used red mud (RM), SiC, pore-forming agents, and catalysts to fabricate a porous silicon carbide (SiC) ceramic filter via sintering at 1000 °C, achieving a bending strength of 65.36 MPa and a porosity of 30.15 vol.%, with good oil removal performance. Tong Lv et al. [10] used mechanical–thermal activation to convert dredged sludge into a supplementary cementitious material. Wang J et al. [11] stabilized electroplating sludge rich in heavy metals using low-carbon ternary cement. The formation of additional hydration products and various calcium aluminate phases through pozzolanic reactions improved the physical encapsulation of heavy metal ions, offering a reference approach for hazardous waste disposal. Xiaobing Ma et al. [12] added 5% calcium carbide residue and 15% gypsum to sewage sludge ash generated during incineration, achieving a compressive strength of 53 MPa after 28 days of curing. Waste concrete sludge has also been explored as an alkali source for recovering reactive MgO from waste brine [13], and after screening, the filtered residue can be reused as coarse and fine aggregates in concrete production [14,15].

Limestone is widely recognized as a valuable auxiliary material in steel and metallurgical processes [16], a primary raw material for sand and gravel aggregates [17], and is also used in flue gas desulfurization and denitrification in blast furnaces [18]. When mixed into soil, limestone waste can reinforce subgrades, improving both soil strength and resistance to deformation [19]. Large volumes of waste residue are generated during limestone mining. Due to the relatively low CaO content in this waste, it can be used to produce low-lime, low-carbon clinker [20]. Waste limestone powder, slag powder, and fly ash can be blended to form ternary composite cementitious materials, which are used to bind coal gangue into green, low-carbon backfill materials [21,22]. Limestone waste, when combined with fly ash, has also been applied to treat acid mine drainage (AMD) generated from coal mining, leveraging its high adsorption capacity [23]. Partial replacement of cement with 5–20% limestone waste micropowder in pervious concrete does not significantly reduce compressive strength but enhances the immobilization and adsorption of heavy metal ions within the material [24]. Some researchers have used limestone powder as a supplementary cementitious material in concrete. While increasing its replacement ratio for fly ash can lead to a decrease in compressive strength, it can improve shrinkage resistance, freeze–thaw durability, and overall long-term performance [25]. When the specific surface area is ≤600 m^2^/kg, the recommended content should not exceed 15%, as excessive dosage can significantly reduce both compressive and bond strength with reinforcement. Conversely, increasing fineness can significantly improve both properties [26].

Cementitious materials, including fly ash, ground granulated blast furnace slag, silica fume, and limestone powder, have been widely explored as supplementary cementitious materials (SCMs) due to their ability to enhance concrete performance and reduce cement consumption. Among these, finely ground limestone powder has been recognized for its physical filler effect, nucleation ability, and chemical interaction with aluminates to form carboaluminate phases, which contribute to early strength and improved microstructure. Despite these advances, most research, to date, has focused on general limestone waste, while little attention has been paid to the dust filter residue (sludge) generated during limestone mining operations. Although LMDFR exists in the form of sludge and poses challenges for ecological disposal, it is essentially composed of fine limestone particles. From a technical standpoint, its application as a supplementary cementitious material (SCM) in concrete is feasible. From the perspective of materials science, this study advances the understanding of LMDFR sludge as a viable powder-based material. In the field of civil engineering, it promotes the utilization of industrial solid waste in concrete production. Environmentally, converting LMDFR sludge from waste into a resource helps reduce its ecological impact. Economically, whereas previous disposal of LMDFR sludge required substantial financial and labor input, its transformation into a high-value SCM introduces economic value and generates tangible economic benefits.

This study focuses on the dust filter residue—a bulk industrial solid waste generated from limestone mining in Panzhihua City. The material properties of the residue were analyzed, and its feasibility as a concrete admixture was systematically evaluated. Cement mortars were prepared using composite admixtures in which FA was replaced by the residue at substitution rates of 0%, 25%, 50%, 75%, and 100%. Flowability of fresh mortar was tested, followed by the determination of flexural and compressive strengths at 7 and 28 days of curing. Additionally, XRD, SEM, TG, and DSC analyses were conducted on samples cured for 28 days. This research provides a technical support for the industrial-scale utilization of LMDFR as a supplementary cementitious material in concrete. The work carries significant economic value and social benefits in the context of bulk solid waste management.

## 2. Material Analysis and Test Plan Design

### 2.1. Test Materials

#### 2.1.1. LMDFR

**Figure 1:**
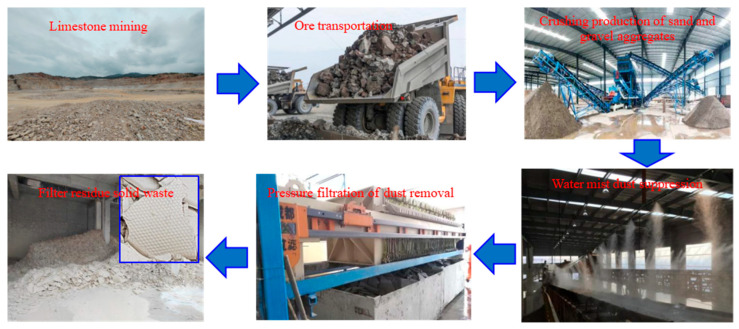
Process flow diagram of dust removal filter residue generation in limestone mining.

The LMDFR used in this experiment was sourced from the Longdong Limestone Mine of Jinruida New Building Materials Co., Ltd., located in Panzhihua City, China. As shown in Figure 1, during the crushing and processing of limestone into sand and gravel aggregates, a significant amount of dust is generated. A water mist spray system is employed for dust suppression, which produces sludge containing a high concentration of fine limestone particles. This sludge is treated through pressure filtration to recover water for reuse in the production process. The resulting solid waste—the LMDFR—is the subject of this study. The annual generation of this residue is approximately 300,000 tons.

The moisture content of the LMDFR was determined to be 18.71% using the oven-drying method. After drying and dispersion, X-ray diffraction (XRD) and X-ray fluorescence (XRF) analyses were conducted.

**Figure 2:**
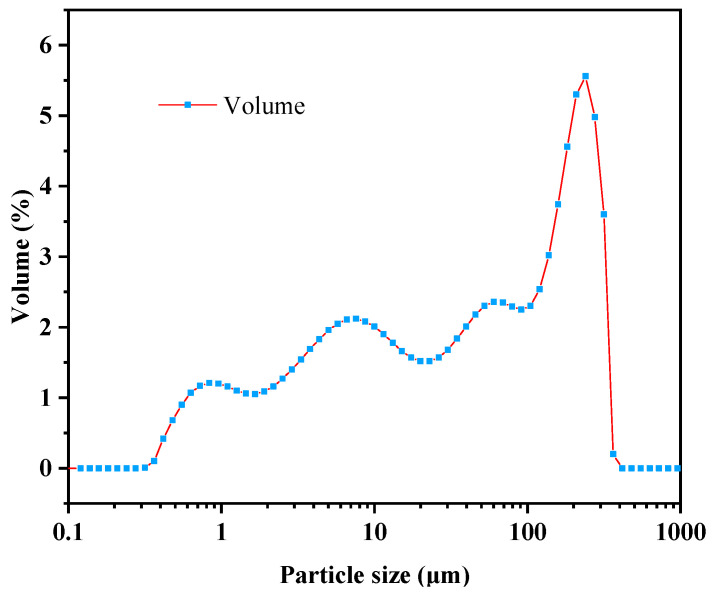
Particle size distribution of LMDFR.

Laser particle size analysis was carried out using a Hydro 2000MU (A) instrument (Malvern, Worcestershire, UK), in accordance with the standard particle size analysis–laser diffraction methods (GB/T 19077-2016/ISO 13320:2009) [27], and the results are shown in Figure 2. The specific surface area of the residue was measured to be 1.3 m^2^/g. The surface-area mean particle diameter D [3,2] was 4.624 μm, while the volume mean diameter D [4,3] was 82.630 μm. Particles with diameters less than 1.430, 38.576, and 232.562 μm accounted for 10%, 50%, and 90% of the sample, respectively.

The specific gravity of the LMDFR was determined using the Le Chatelier flask method, following the Standard for Geotechnical Testing Method (GB/T 50123-2019) [28]. The measured specific gravity was 2.694 (based on pure water at 4 °C), which is approximately equivalent to that of conventional FA.

#### 2.1.2. FA

The FA used in this experiment was Class II FA produced by Liyuan FA Products Co., Ltd. in Panzhihua, China [14]. It appeared dark gray in color, and its main performance indicators are listed in Table 1. The material meets the technical requirements for Class II FA used in concrete, as specified in GB/T 1596-2017 (China) [29].

#### 2.1.3. Other Test Materials

The mixing water used in the experiment was municipal tap water. The cement employed in the cement mortar tests was reference cement, specifically the standard reference cement for concrete admixture testing (P·I 42.5 Portland cement) produced by China United Cement Corporation. The main chemical composition of the cement is shown in Table 2 [14], and its physical properties are listed in Table 3 [14].

**Figure 3:**
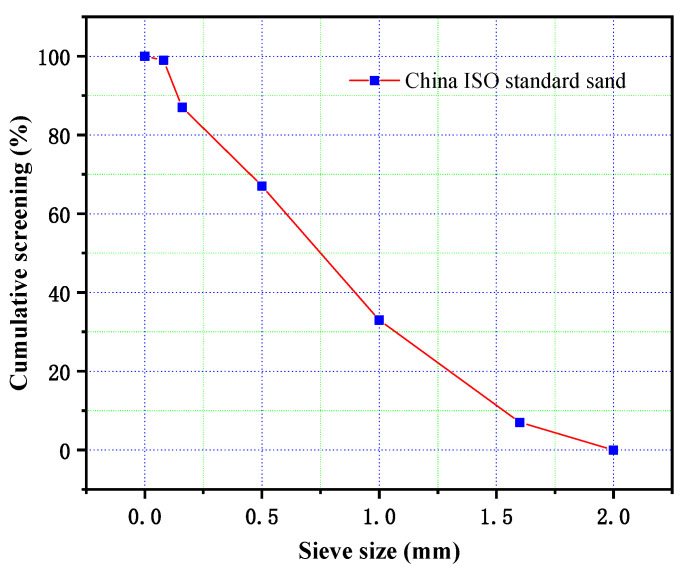
Particle size distribution of ISO standard sand.

The sand used in the experiment was “China ISO Standard Sand”, which is the legally designated reference material equivalent to the international standard ISO 679 for testing the strength of cement. The main performance parameters are listed in Table 4, and the particle size distribution is shown in Figure 3 [14]. 

### 2.2. Test Plan Design and Method

Cement mortar specimens were prepared using LMDFR and Class II FA under the following conditions: no admixture, FA only, LMDFR only, and composite admixtures with LMDFR-to-FA ratios of 1:3, 1:1, and 3:1, respectively. The flowability ratio of the cement mortar was measured in accordance with GB/T 2419-2024: Test Method for Fluidity of Cement Mortar [30]. Specimens were then prepared and cured under standard conditions. The 7- and 28-day flexural and compressive strengths, as well as the activity index, were determined following the procedures outlined in GB/T 17671-2021: Test Method of Cement Mortar Strength (ISO Method) [31] and GB/T 51003-2014: Technical Code for Application of Mineral Admixtures [32]. Additionally, thermal analysis (TG-DSC), X-ray diffraction (XRD), and scanning electron microscopy (SEM) were performed to analyze the mineralogical and microstructural characteristics of the specimens. The experimental design and mixture proportions are summarized in Table 5.

XRD Analysis: X-ray diffraction (XRD) was performed using an XPert PRO diffractometer (PANalytical, Almelo, The Netherlands) operating at 40 kV and 40 mA. The scan was conducted in Gonio mode with a continuous scan type, a step size of 0.0260°/min, and Cu Kα radiation (λ = 1.5406 Å). Peak identification and fitting were performed using Gaussian functions.

SEM and EDS Analysis: Scanning electron microscopy (SEM) was conducted using a ZEISS EVO 180 microscope (ZEISS, Oberkochen, Germany) equipped with a BRUKER XFlash 6130 energy-dispersive X-ray spectroscopy (EDS) detector (BRUKER, Karlsruhe, Germany). Imaging was performed at an accelerating voltage of 20 kV, using both secondary electron (SE) and back-scattered electron (BSE) modes to capture surface morphology and compositional contrast. All samples were gold-coated prior to imaging to improve conductivity.

## 3. Flowability and Hydration Activity Test Analysis

### 3.1. Fluidity

The flowability of the cement mortar was measured according to the Test Method for Fluidity of Cement Mortar (GB/T 2419-2024). During specimen preparation, a damp cloth was used to wipe the surface of the flow table, the inner wall of the truncated cone mold, the tamping rod, and all tools that would come into contact with the mortar. The mold was placed at the center of the flow table and covered with a damp cloth. The freshly mixed mortar was quickly filled into the mold in two layers. The first layer was filled to approximately two-thirds of the mold height. A knife was used to score the surface five times in two perpendicular directions, followed by 15 uniform tamps with the rod from the edge toward the center. The second layer was then added, filled to about 20 mm above the top of the mold. The surface was again scored five times in perpendicular directions, followed by 10 tamps from the edge to the center. After tamping, the mortar should be slightly above the top of the mold. The tamping depth for the first layer should reach half of its height, while for the second layer, it should not exceed the surface of the compacted first layer. After tamping, the mold collar was removed. Using a knife held at a shallow angle, the excess mortar above the mold was scraped off from the center toward the edges in two smooth passes. Any mortar that fell onto the table surface was also cleaned off. The truncated cone mold was then gently lifted vertically, and the flow table was immediately activated at a rate of one drop per second, completing 25 drops within 25 ± 1 s. The entire procedure—from water addition to the measurement of the final spread diameter—was completed within 6 min.

**Figure 4:**
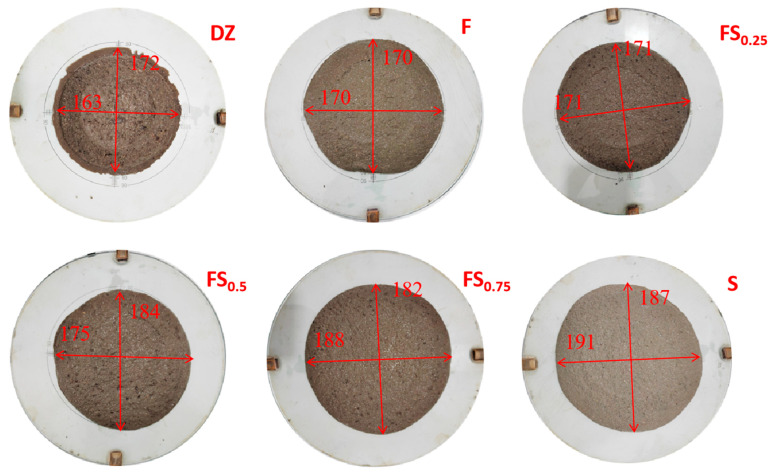
Fluidity condition.

Upon completion, the flow diameter of the mortar was measured in two perpendicular directions across the base using calipers, and the average value was recorded as the flowability. The effect of different replacement ratios of LMDFR on the flowability of cement mortar is shown in Figure 4, and the detailed test results are provided in Table 6.

The average flowability of the control group (without any admixture) was 168 mm. When only FA was added, the average flowability increased slightly to 170 mm, corresponding to a relative flowability ratio of 101.19%. For the composite admixture of LMDFR and FA: at a 1:3 ratio (LMDFR to FA), the average flowability was 171 mm (101.79%); at a 1:1 ratio, it reached 180 mm (106.85%); at a 3:1 ratio, the value further increased to 185 mm (110.12%). When only LMDFR was used, the average flowability reached a maximum of 189 mm, representing 112.50% of the control group.

These results indicate that incorporating LMDFR can significantly improve the flowability of cement mortar, with the best performance observed when used alone. In contrast, the lowest flowability was recorded for the mortar containing only FA, which is consistent with the SEM microstructural observations. The tested FA contains a large proportion of hard, irregularly shaped particles, which negatively affect the workability of the mixture. In the composite admixtures, increasing the proportion of LMDFR leads to a progressive enhancement in flowability, demonstrating its positive effect on improving the rheological performance of cement mortar.

### 3.2. Flexural Strength, Compressive Strength, and Activity Index

The flexural strength, compressive strength, and activity index of cement mortars incorporating different proportions of LMDFR–FA composite admixtures were evaluated in accordance with GB/T 17671-2021: Test Method of Cement Mortar Strength (ISO Method) and GB/T 51003-2014: Technical Code for Application of Mineral Admixtures. The cement mortar specimens were cast in molds measuring 40 mm × 40 mm × 160 mm and were cured under standard conditions in a temperature- and humidity-controlled chamber at 20 ± 1 °C and relative humidity above 95% for 7 and 28 days, respectively. The flexural and compressive strength tests were carried out using a YAW-1000D electric compression-flexure testing machine, manufactured by Beijing Sanyu Weiye Testing Machine Co., Ltd. (Beijing, China).

According to the Chinese national standard GB/T 17671-2021 “Test Method of Cement Mortar Strength (ISO Method), Cement and Concrete Composites”, each set of cement mortar specimens consists of three prismatic samples (40 mm × 40 mm × 160 mm). The flexural strength of each group is determined by averaging the three measured values; if any value deviates by more than ±10% from the average, it should be excluded, and the average of the remaining two values is taken as the final result.

The compressive strength is determined using six test values per group. The arithmetic mean of the six values is used as the result; if one value deviates by more than ±10% from the mean, it should be discarded, and the average of the remaining five values is used. If among these five values there is still any that deviates more than ±10% from their average, the result of this group is deemed invalid.

The results for flexural strength, compressive strength, and activity index are summarized in Table 7.

After the addition of admixtures, both the 7- and 28-day flexural and compressive strengths of cement mortar showed varying degrees of reduction compared with the control group. When only FA was added, the 7-day flexural and compressive strengths were 84.62% and 69.82% of the control group, respectively, while the 28-day strengths were 85.14% and 71.52%, respectively. When composite admixtures of LMDFR and FA, or only filter LMDFR, were added, the 7-day flexural strength of the mortar was approximately equal to that of the FA-only group. However, the 7-day compressive strength was significantly higher than that of the FA-only group. The 7-day activity indices of groups F, FS_0.25_, FS_0.5_, FS_0.75_, and S were 94.63%, 99.57%, 79.80%, 87.47%, and (S), respectively, representing increases of 24.81%, 29.67%, 9.98%, and 17.65% compared with the FA group (F). These results indicate that the addition of LMDFR imparts a notable early-strength effect, with the highest 7-day strength observed at a 1:1 LMDFR-to-FA ratio. At 28 days, the flexural strength of mortars with composite or single LMDFR admixtures significantly increased compared with their 7-day values, similar to the trend observed in the control group (DZ), and also showed improvements over the FA-only group (F). However, the 28-day compressive strength generally exhibited a declining trend relative to the corresponding 7-day values. The 28-day activity indices for groups F, FS_0.25_, FS_0.5_, FS_0.75_, and S were 71.52%, 67.04%, 74.66%, 83.18%, and 61.21%, respectively, indicating an overall decrease of about 25% in strength compared with 7-day values. In particular, when only LMDFR was used, the 28-day activity index dropped to 61.21%, which is significantly lower than that of the FA-only group (F, 71.52%).

The LMDFR primarily contributes through a physical filling effect [33,34]. In addition, limestone particles can adsorb Ca^2+^ ions released during the hydration of tricalcium silicate (C_3_S), thereby reducing the accumulation and oriented crystallization of calcium hydroxide (CH) at the interface and increasing the content of C–S–H gel, while also providing nucleation sites for hydration products [35,36,37]. This nucleation effect can accelerate and enhance the hydration of silicate minerals in cement, promoting the early growth and precipitation of hydration products [38]. Furthermore, CaCO_3_ in the LMDFR reacts with C_3_A (3CaO·Al_2_O_3_) in cement during hydration to form hemicarbonate (Hc), a metastable calcium carboaluminate phase (as shown in Equation (1)). Over time, Hc gradually transforms into monocarbonate (Mc) (as shown in Equation (2)) [39,40], helping to refine the pore structure. Based on these mechanisms, the 7-day strength of cement mortar containing LMDFR is comparable with or slightly lower than that of the reference group.

C_3_A + 0.5CaCO_3_ + 12H_2_O → C_3_A∙0.5CaCO_3_∙12H_2_O (1)

2C_3_A∙0.5CaCO_3_∙12H_2_O + CaCO_3_ → 2C_3_A∙CaCO_3_∙11H_2_O + H_2_O (2)

The observed decline in 28-day strength after the incorporation of LMDFR can be attributed to several factors: First, the early-formed hemicarbonate (Hc) may fail to stably convert into monocarbonate (Mc), leading to a loose microstructure [41]. Second, excessive CaCO_3_ tends to accumulate at the aggregate–paste interface, forming a weak interfacial transition zone. Additionally, when ambient humidity falls below 90%, the reaction between limestone powder and C_3_A is hindered, resulting in a stagnation of late-stage hydration. Due to the low reactivity of limestone powder, only a small fraction participates in hydration reactions. Replacing cement with limestone powder reduces the total amount of cementitious material, which, in turn, decreases the quantity of hydration products per unit volume of cement-based material. This is commonly referred to as the dilution effect. When the replacement level exceeds 20%, excess CaCO_3_ further dilutes the active cement components [35,42,43,44], leading to insufficient hydration at later stages. Moreover, coarse limestone particles (with a specific surface area < 350 m^2^/kg) are generally inert and function only as fillers without significantly participating in hydration reactions [34].

In contrast, FA contains reactive SiO_2_ and Al_2_O_3_, which can react with calcium hydroxide (CH) to form calcium aluminosilicate hydrates (C–A–S–H), contributing to the development of late-stage strength [45]. Therefore, the 28-day strength of the FA-only group (F) is significantly higher than its 7-day strength.

## 4. Microscopic Analysis

### 4.1. SEM Analysis of Test Materials and Cement Mortar Specimens

#### 4.1.1. SEM Analysis of Test Materials

(a) SEM Analysis of LMDFR

**Figure 5:**
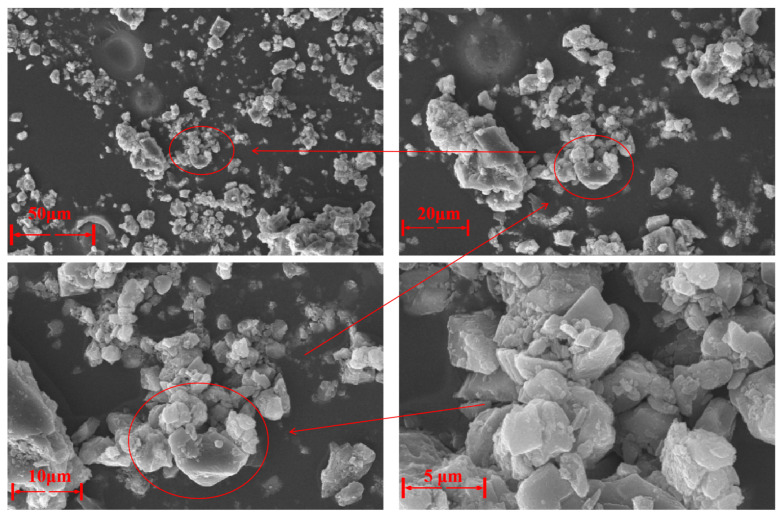
SEM microscopic morphology of LMDFR.

As shown in Figure 5, the SEM images of the LMDFR under different magnifications reveal microstructural features that are consistent with the macroscopic observations. The particles exhibit irregular shapes and a wide size distribution, with only a few needle-like or flake-like particles observed. Some degree of particle agglomeration is also present. At a minimum resolution of 10 μm, the particles appear as unevenly sized, block-like granules smaller than 10 μm, with smooth surfaces, no visible cracks, and colors ranging from white to light yellow. These fine particles indicate good dispersibility and filling capability. At a higher resolution of 2 μm, the limestone filter residue particles are observed to have smooth, crack-free surfaces, further confirming their favorable morphology. From Figure 5 it can also be observed that the LMDFR is not particularly dense in texture, and the mineral phases display a layered structural distribution, which is consistent with the specific gravity measured by the Le Chatelier flask method.

**Figure 6:**
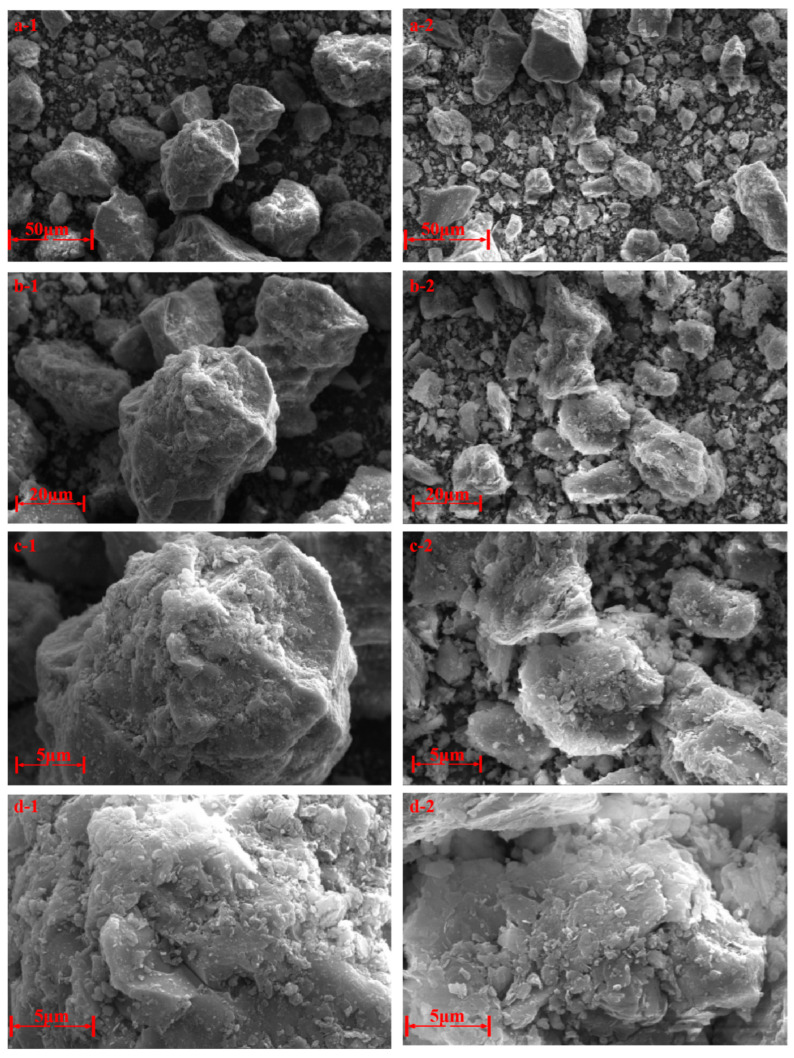
SEM pattern of FA.

Scanning electron microscopy (SEM) was used to observe the microstructure of the Class II FA used in the experiment, as shown in Figure 6. It can be seen from the images that the FA primarily consists of irregularly shaped particles. Both large and small particles are present in significant proportions, while medium-sized particles are relatively scarce, indicating a discontinuous particle size distribution.

Two main types of particles can be identified: (1) The first type, shown in Figure 6(a-1,b-1,c-1,d-1), is mainly composed of large particles. These particles are dense, have poor particle size continuity, and contain a high proportion of coarse material, which results in poor grindability. (2) The second type, shown in Figure 6(a-2,b-2,c-2,d-2), is primarily composed of small particles. These particles are generally less dense; exhibit better particle size continuity compared with the first type; and, under higher magnification, display layered, flaky surface morphology. The surface appears relatively rough, which contributes to better grindability.

#### 4.1.2. SEM Analysis of Cement Mortar Specimens

**Figure 7:**
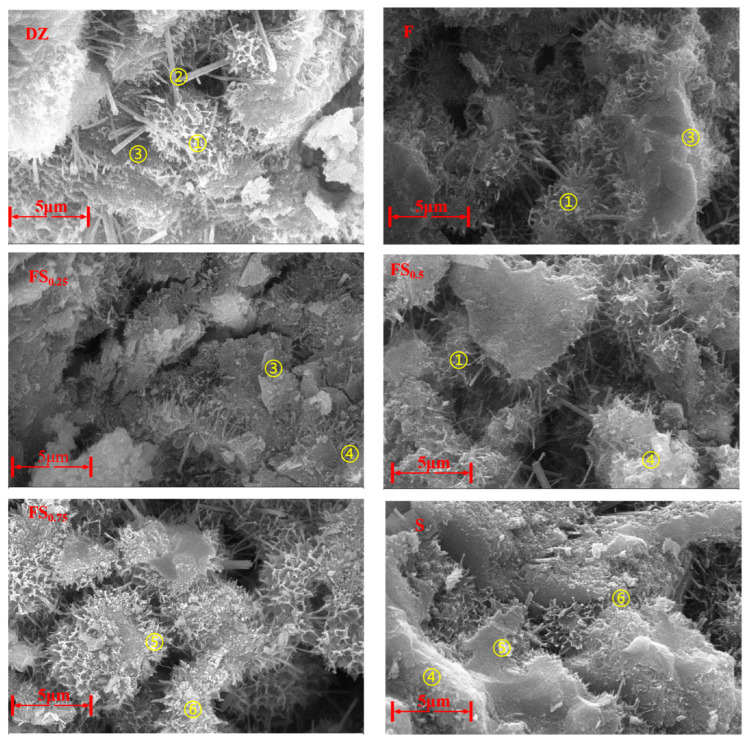
SEM microstructure of cement mortar cured for 28 days. (① C-S-H, ② AFt, ③ CH, ④ Mc, ⑤ C-A-S-H, ⑥ CaCO_3_).

Scanning electron microscopy (SEM) was performed on cement mortar specimens prepared according to the mix proportions in Table 5 and cured for 28 days. The analysis was conducted at a magnification of 5000× (scale bar = 5 μm), with an accelerating voltage of 20 kV and a working distance of 7–10 mm. The microstructure of hydration products and pore morphology is shown in Figure 7.

Based on the correlation between the SEM images and the phase compositions of each group, the following observations can be made:

(1) DZ group (pure cement, control group): A dense network of fibrous C–S–H (Point ①) gel is interwoven with dispersed plate-like Ca(OH)_2_ (Point ③) crystals (CH). Clusters of needle-like ettringite (AFt) (Point ②) with diameters around 100–200 nm are also visible. These observations are consistent with the strong C–S–H and AFt peaks in XRD patterns and the prominent CH decomposition peak at 400–500 °C in TG curves, corresponding to the plate-like CH crystals.

(2) F group (FA only): Spherical FA particles (1–5 μm) are partially coated with C–S–H gel, and CH crystals are noticeably reduced. Some FA particles remain unreacted, as indicated by their smooth surfaces. In agreement with XRD and TG-DSC results, the CH peak weakens and the SiO_2_ peak intensifies in the XRD pattern (due to residual quartz from unreacted FA and the cement mortar). The CH decomposition peak in TG also shows reduced area, confirming pozzolanic reaction consumption of CH.

(3) FS composite groups (LMDFR + FA): In groups FS_0.25_ and FS_0.5_, C–S–H gel coexists with plate-like monocarbonate (Mc, Point ④) phases, and overall porosity is reduced. In the FS_0.75_ group, the Mc plate structures become denser, with a small amount of unreacted angular limestone (Point ⑥) particles still observed. This correlates well with the XRD and TG-DSC results: the Mc peak intensity increases with the limestone content in XRD, and mass loss at 100–200 °C in TG increases due to interlayer water loss from Mc.

(4) S group (LMDFR only): A large amount of plate-like Mc (Point ④) and flocculent C–A–S–H (Point ⑤) gel is present, with virtually no CH crystals observed. In some regions, zeolite-like calcium aluminosilicate hydrates (Ca_2_(Si_9_Al_3_)O_24_·8H_2_O) appear in the form of cubic crystal clusters. These findings align with the XRD and TG-DSC results: distinct peaks for Mc and zeolite phases are observed in XRD, while the CH peak disappears. The CH decomposition peak is nearly absent in the TG curve.

### 4.2. TG and DSC Analysis of Cement Mortar

**Figure 8:**
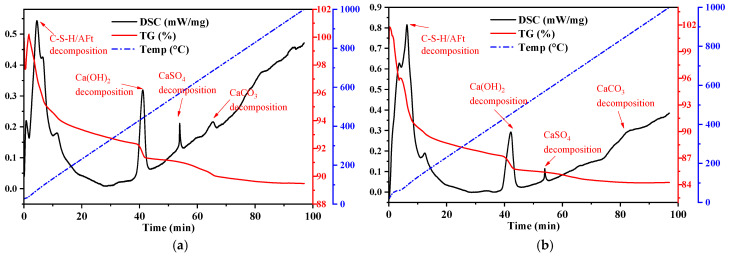

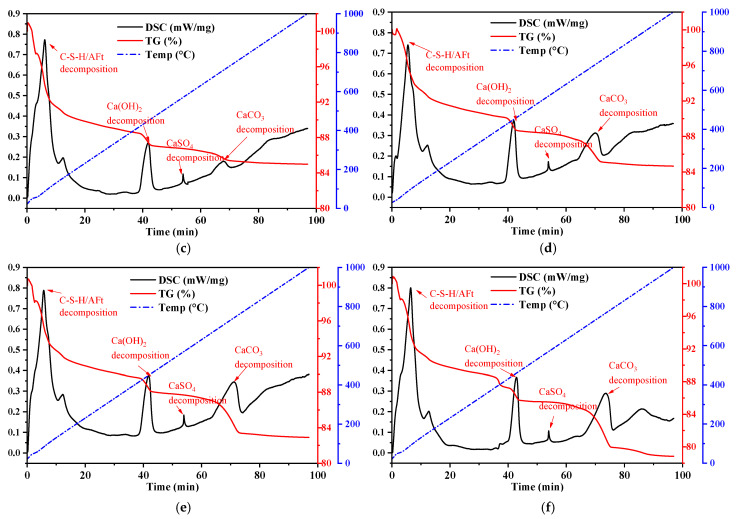
TG-DSC thermal analysis curves of cement mortar cured for 28 days; (**a**) DZ, (**b**) F, (**c**) FS_0.25_, (**d**) FS_0.5_, (**e**) FS_0.75_, and (**f**) S.

Thermogravimetric (TG) and differential scanning calorimetry (DSC) analyses were conducted on cement mortar specimens prepared according to the mix proportions shown in Table 5 and cured for 28 days. The TG and DSC curves are presented in Figure 8.

The thermal analysis curves of the cement mortar hydration products are shown in Figure 8. The right vertical axis represents the TG curve, indicating mass percentage (%), while the left vertical axis represents the DSC curve, which reflects the heat flow rate per gram of sample (mW/mg)—that is, the differential scanning calorimetry signal normalized by sample mass. The TG curve reveals the stages of mass loss associated with the decomposition of hydration products such as C–S–H, Ca(OH)_2_ (CH), and ettringite (AFt). The endothermic peaks in the DSC curve correspond to the heat absorption of these decomposition reactions, while exothermic peaks may indicate crystalline transformations or oxidation reactions. As illustrated in the figure, the thermal analysis was performed at a heating rate of 10 °C/min, with the sample reaching 1000 °C in 100 min.

Cement mortars containing different proportions of limestone mine dust filter residue exhibited generally similar TG and DSC profiles. In the first 2 min, fluctuations in the TG curve were observed due to air or residual impurities in the crucible; this portion was disregarded. At 6–8 min (corresponding to 60–80 °C), a significant drop in the TG curve appeared due to the decomposition of interlayer water, C–S–H gel, and AFt, accompanied by the first endothermic peak in the DSC curve. As the temperature continued to rise, the decomposition of hydration products progressed. Around 12 min, the TG curve showed a continued gradual decline, while the DSC curve exhibited the second endothermic peak. At 38–42 min (approximately 400 °C), the release of crystalline and chemically bound water and the dehydration of Ca(OH)_2_ caused a sharp drop in the TG curve. This corresponded to a sharp rise in the DSC curve, reflecting an endothermic event. After the complete dehydration of Ca(OH)_2_, the heat flow rate decreased, and the DSC curve returned to its upward trend. At 54–56 min (around 540–560 °C), another DSC peak appeared without a notable mass loss in the TG curve, which may be attributed to the decomposition of residual gypsum in the mortar (as shown in Equation (3)). Finally, between 66 and 78 min, as the temperature stabilized at 660–780 °C, a clear inflection point appeared in the TG curve along with a corresponding DSC peak, indicating the thermal decomposition of CaCO_3_.

(3) $$Ca{SO}_{4}\cdot 2H_{2}O\overset{540-560{}^{\circ}C}{\to }Ca{SO}_{4}+2H_{2}O↑$$

Throughout the heating process, the total mass loss observed for each group was as follows: DZ group (control)—approximately 10.5%; F group (FA only)—15.8%; FS_0.25_—15.0%; FS_0.5_—15.4%; FS_0.75_—19.0%; and S group (LMDFR only)—21.0%. The greater the proportion of LMDFR in the composite admixture, the more residual CaCO_3_ remains in the cement mortar. As a result, the mass loss near 700 °C—associated with CaCO_3_ decomposition—becomes more pronounced.

When the LMDFR is blended with FA, it contributes to the formation of calcium carboaluminate hydrates (such as ${{[\mathrm{Ca}}_{2}{\mathrm{Al}(\mathrm{OH})}_{6}]}^{+}\cdot {\left[{\mathrm{CO}}_{3}^{2-}\right]}_{0.5}\cdot nH_{2}O$ and ${{[\mathrm{Ca}}_{2}{\mathrm{Al}(\mathrm{OH})}_{6}]}^{+}\cdot {\mathrm{CO}}_{3}^{2-}\cdot {3H}_{2}O$) and C–A–S–H gels, resulting in increased mass loss in the 50–200 °C range. However, it also suppresses the formation of Ca(OH)_2_, as evidenced by the weakened endothermic peak near 450 °C. In the S group (LMDFR only), a significant amount of unreacted CaCO_3_ leads to marked mass loss between 600 and 800 °C. The F group (FA only) shows a strong pozzolanic reaction, significantly consuming Ca(OH)_2_. When LMDFR is co-blended with FA, the Ca(OH)_2_ content decreases even further. In the FS_0.75_ group, the Ca(OH)_2_ peak almost completely disappears.

Among the composite admixtures, the FS_0.5_ group (1:1 ratio) appears to achieve the best synergistic effect: moderate mass loss in the 50–200 °C range indicates the stable formation of C–S–H and AFt. A relatively low mass loss at 600–800 °C suggests no excessive residual CaCO_3_, indicating better material balance and hydration efficiency.

### 4.3. XRD Analysis of Test Materials and Cement Mortar

#### 4.3.1. XRD Analysis of Test Materials

**Figure 9:**
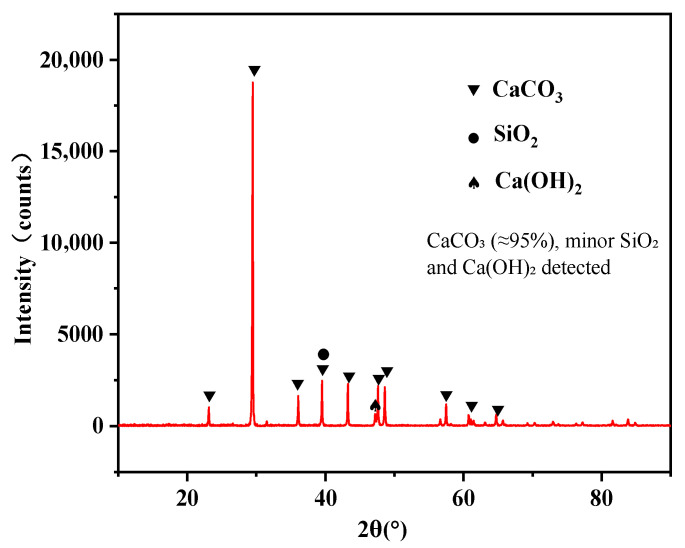
XRD pattern of LMDFR.

The LMDFR was characterized using X-ray diffraction (XRD) to determine its mineralogical composition, and X-ray fluorescence (XRF) was employed to analyze its chemical composition. The results showed that the CaCO_3_ content of the residue exceeded 95%; the remaining 5% consists of small amounts of a mixture of MgO, Al_2_O_3_, SiO_2_, P_2_O_5_, TiO_2_, and Fe_2_O_3_, as illustrated in Figure 9.

**Figure 10:**
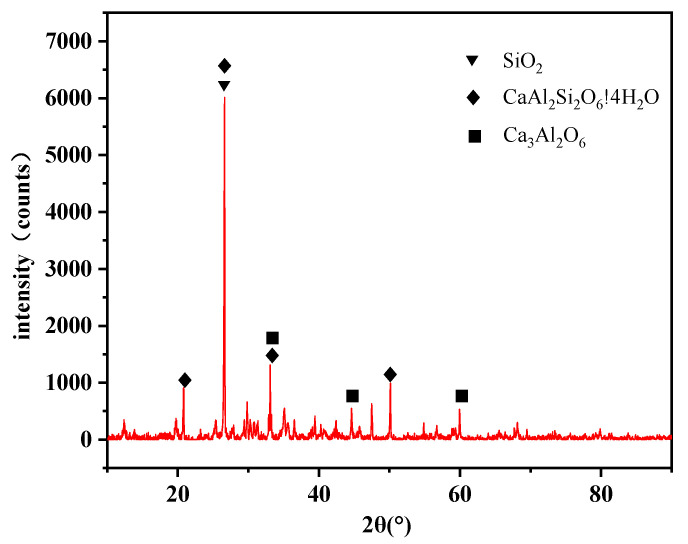
XRD pattern of FA.

X-ray diffraction (XRD) analysis was performed on the FA, and the diffraction pattern is shown in Figure 10. A prominent peak at 2θ = 26.6° indicates the presence of quartz (SiO_2_), an inert component whose excessive content may reduce pozzolanic activity. Peaks at 2θ = 22.1°, 33.2°, and 51.8° correspond to hydrated calcium aluminosilicate (CaAl_2_Si_2_O_8_·4H_2_O), which contributes to early strength development and enhances resistance to sulfate attack during the hydration process. Additionally, peaks at 2θ = 33.2°, 47.6°, and 62.3° indicate the presence of tricalcium aluminate (Ca_3_Al_2_O_6_), a highly reactive component that can rapidly hydrate and may cause flash setting under certain conditions.

#### 4.3.2. XRD Analysis of Cement Mortar

**Figure 11:**
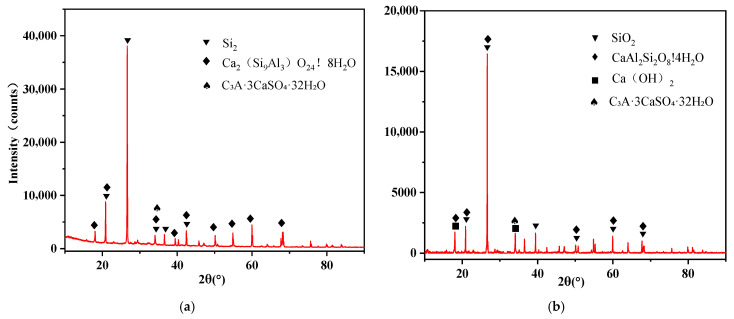

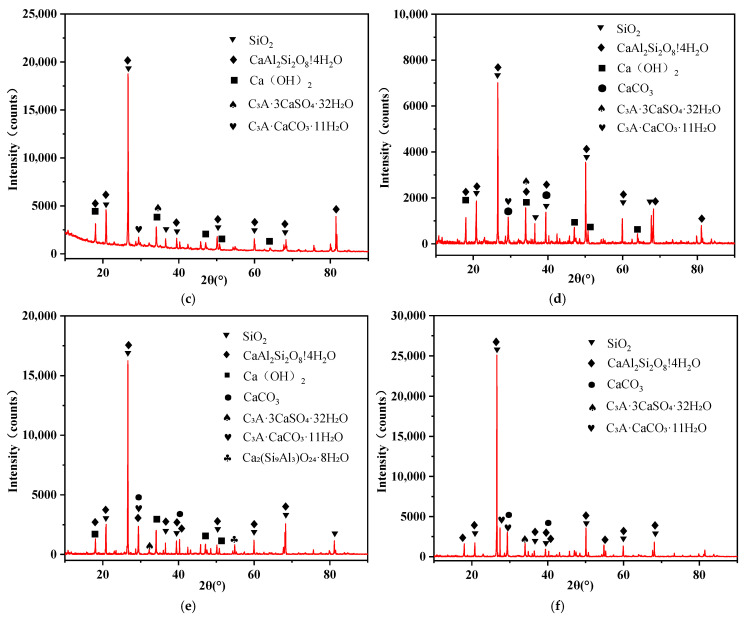
XRD patterns of cement mortar specimens cured for 28 days; (**a**) DZ, (**b**) F, (**c**) FS_0.25_, (**d**) FS_0.5_, (**e**) FS_0.75_, and (**f**) S.

X-ray diffraction (XRD) analysis was performed on cement mortar specimens prepared according to the mix proportions shown in Table 5 and cured for 28 days. The resulting XRD patterns are presented in Figure 11.

Comparative analysis of the characteristic XRD patterns for each group yields the following observations: In the DB group (without any admixture), broad peaks corresponding to C–S–H, as well as distinct signals for ettringite (AFt) and quartz (SiO_2_) from the mortar, were detected. This represents typical cement hydration behavior without interference from mineral admixture. In the F group (FA only), peaks corresponding to Ca(OH)_2_, SiO_2_, AFt, and C–S–H were identified. The pozzolanic reaction of FA consumes Ca(OH)_2_ and contributes to the formation of additional C–S–H gel. In the FS composite groups (LMDFR + FA), monocarbonate (Mc) peaks were prominent, while the Ca(OH)_2_ peaks weakened further, indicating that the limestone powder promoted the formation of carboaluminate phases and suppressed Ca(OH)_2_ accumulation. Starting from the FS0.5 group, where the residue accounts for ≥50% of the composite admixture (i.e., 15% of the total cementitious material), residual unreacted limestone powder becomes clearly detectable. In the S group (LMDFR only), strong monocarbonate (Mc) peaks were observed, along with characteristic peaks of zeolite-like calcium aluminosilicate hydrate (Ca_2_(Si_9_Al_3_)O_24_·8H_2_O) near 2θ = 50–55°. Virtually no Ca(OH)_2_ was detected, suggesting that the limestone powder completely inhibited Ca(OH)_2_ accumulation and promoted the formation of aluminosilicate-rich phases.

The mass loss in the 100–200 °C range, as shown in the TG curves, is consistent with the interlayer water loss of C–S–H and monocarbonate (Mc) identified by XRD. The mass loss at 400–500 °C, corresponding to Ca(OH)_2_ decomposition, is positively correlated with the intensity of the Ca(OH)_2_ peaks in XRD.

## 5. Conclusions

In this study, the material properties of two types of industrial solid waste—LMDFR and FA—were investigated. Building on the authors’ previous research, cement mortar specimens were prepared by replacing FA with LMDFR at replacement ratios of 0%, 25%, 50%, 75%, and 100%, forming composite admixtures to replace 30% of the cement. The workability, hydration activity, and feasibility of using the LMDFR as a supplementary cementitious material were assessed. In addition, TG, XRD, and SEM analyses were performed on 28-day cured specimens to study hydration behavior and microstructural mechanisms. LMDFR meets the relevant technical requirements for the application of limestone powder as a mineral admixture in concrete, as specified in the Chinese national standard GB/T 51003-2014 Technical Code for Application of Mineral Admixtures [32]. The specific conclusions are as follows:

(1) The LMDFR primarily consists of CaCO_3_ with a purity over 95%, with a specific surface area of approximately 1.3 m^2^/g, surface-area mean particle diameter D [3,2] = 4.624 μm, volume mean particle diameter D [4,3] = 82.630 μm, and a specific gravity of 2.694. The particles are irregular in shape, vary in size, exhibit layered mineral structure, and are not particularly dense.

(2) When replacing 30% of cement with composite admixtures of LMDFR and FA at various ratios, the flowability of cement mortar improved in all cases compared with the control group. Flowability increased with higher LMDFR content. Specifically, the flowability of FA-only mortar was 101.76%, while that of mortar with only LMDFR reached 112.83%, consistent with the physical properties of the LMDFR.

(3) The incorporation of LMDFR promotes the conversion of hemicarbonate to monocarbonate during the early hydration reaction of cement mortar, which contributes to the increase in early compressive strength. Replacing 30% of cement with FA, LMDFR, or their combinations generally led to a reduction in flexural and compressive strength due to reduced cement content. The addition of LMDFR had little effect on flexural strength at 7 and 28 days, though 28-day flexural strength was consistently higher. In contrast, 7-day compressive strength improved with higher residue content, but 28-day compressive strength decreased. For example, 30% FA replacement yielded 69.82% (7 days) and 71.52% (28 days) of the control compressive strength, while 30% LMDFR replacement resulted in 87.47% (7 days) and 61.21% (28 days). The highest compressive strength (99.63% (7 days) and 83.18% (28 days) of control) was achieved when FA and LMDFR were blended 1:1 and used to replace 30% of the cement.

(4) Thermal analysis (TG/DSC) and SEM microstructure observations of 28-day cured mortars showed clear patterns corresponding to the admixture ratios. These results were consistent with the trends observed in compressive strength performance, verifying the correlation between microstructural evolution and mechanical behavior.

To further promote the comprehensive utilization of LMDFR as a supplementary cementitious material in concrete, we suggest that our team or other researchers continue to investigate the durability of concrete incorporating limestone micropowder, its interaction with reinforcement, and the structural performance of the resulting reinforced concrete components in future studies. Once a systematic body of research on LMDFR application as a supplementary cementitious material is established, it can further accelerate the industrial application of LMDFR, thereby reducing the environmental pollution pressure caused by sludge solid waste and achieving resource recycling of waste materials. This has significant implications for both environmental protection and economic benefits.

## Figures and Tables

**Table 1:** Performance parameters of the FA used in the experiment.

Test Items (i)	Fineness	Water Requirement Ratio	Loss on Ignition	Moisture Content	SO_3_ Content
Indicator requirements	≤30%	≤105%	≤8%	≤1%	≤3%
Test results	26%	102%	4.1%	0.9%	0.6%
**Test items (ii)**	$f_{CaO}^{-}$	**Density**	**Stability (Reilich method)**	**Intensity activity Index**	**SiO_2_ + Al_2_O_3_ + Fe_2_O_3_**
Indicator requirements	≤1%	≤2.6 g/cm^3^	≤5 mm	≥70%	≥70%
Test results	0.2%	2.4 g/cm^3^	2.1 mm	71.52%	74.5%

**Table 2:** Composition and mass percentage of reference cement %.

Composition	SiO_2_	Al_2_O_3_	Fe_2_O_3_	CaO	MgO	SO_3_	Na_2_Oeq	$f_{CaO}^{-}$	Loss	Cl^−^
Content	22.89	4.58	3.51	64.05	2.15	2.40	0.52	0.90	1.13	0.012

**Table 3:** Physical properties of reference cement.

Fineness	Density	Specific Surface Area	Standard Consistency	Stability (Lei’s Method)	Setting Time		3-Day	
					Initial Setting	Final Coagulation	Flexural Strength	Compressive Strength
0.9%	3.15 g/cm^3^	341 m^2^/kg	24.8%	0.1 mm	140 min	189 min	5.4 MPa	26.2 MPa

**Table 4:** China ISO standard sand main parameter index %.

SiO_2_	Moisture Content	Mud Content	Firing Loss	Cl-	Float Content
>98%	≤0.18%	≤0.18%	<0.47%	≤0.0070%	≤0.0020

**Table 5:** Test scheme and mix ratio %.

Serial Number	Cement /g	Composite Admixture/g		ISO Sand /g	Water /g	Remarks
		FA	LMDFR			
DZ	450 ± 2	0	0	1350 ± 2	225 ± 1	Without admixtures
F	315 ± 1	135	0	1350 ± 2	225 ± 1	FA only
FS_0.25_	315 ± 1	101.25	33.75	1350 ± 2	225 ± 1	LMDFR:FA = 1:3
FS_0.5_	315 ± 1	67.5	67.5	1350 ± 2	225 ± 1	LMDFR:FA = 1:1
FS_0.75_	315 ± 1	33.75	101.25	1350 ± 2	225 ± 1	LMDFR:FA = 3:1
S	315 ± 1	0	135	1350 ± 2	225 ± 1	LMDFR only

**Table 6:** Fluidity test data mm.

NO.	Serial Number	Transverse	Longitudinal	Mean Mobility	Flow Ratio
1	DZ	163	172	168	100.00%
2	F	170	170	170	101.19%
3	FS_0.25_	171	171	171	101.79%
4	FS_0.5_	175	184	180	106.85%
5	FS_0.75_	188	182	185	110.12%
6	S	191	187	189	112.50%

**Table 7:** Flexural strength, compressive strength, and activity index.

Serial Number	Flexural Strength/MPa		Compressive Strength/MPa		Activity Index	
	7-Day	28-Day	7-Day	28-Day	7-Day	28-Day
DZ	6.5	7.4	39.1	44.6	100.00%	100.00%
F	5.5	6.3	27.3	31.9	69.82%	71.52%
FS_0.25_	5.2	7.3	37.0	29.9	94.63%	67.04%
FS_0.5_	5.3	6.9	38.9	37.1	99.49%	83.18%
FS_0.75_	5.6	6.5	31.2	33.3	79.80%	74.66%
S	5.3	6.7	34.2	27.3	87.47%	61.21%

## Data Availability

The original contributions presented in this study are included in the article. Further inquiries can be directed to the corresponding author.
